# Negative Association Between Schizophrenia and Subsequent Cancer Diagnoses—A Retrospective Cohort Study from Germany

**DOI:** 10.3390/ejihpe14120194

**Published:** 2024-11-26

**Authors:** Ira Rodemer, Céline Vetter, Matthias Kalder, André Hajek, Karel Kostev

**Affiliations:** 1 Epidemiology, IQVIA, 60549 Frankfurt, Germany; 2 Department of Gynecology and Obstetrics, Philipps-University of Marburg, 35043 Marburg, Germany; 3 Department of Health Economics and Health Services Research, University Medical Center Hamburg-Eppendorf, 20246 Hamburg, Germany

**Keywords:** schizophrenia, mental disorder, cancer, cancer risk, cancer incidence

## Abstract

Background: Since previous studies have reported contradictory findings regarding the relationship between schizophrenia and cancer, we evaluated the association between schizophrenia and cancer diagnoses. Methods: In this retrospective cohort study, the IQVIA Disease Analyzer database was utilized to examine the incidence of cancer among patients aged over 18 years diagnosed with schizophrenia in German general practices from 2005 to 2022. Patients with schizophrenia were compared with those without the condition, with adjustments made for age, sex, index year of diagnosis, average annual practitioners visit frequency, and comorbidity. Kaplan–Meier curves were used to analyze the 10-year cumulative incidence of schizophrenia and cancer in total amongst patients with and without schizophrenia. Univariate Cox regression analysis was performed to calculate Hazard Ratios (HR) of cancer risk and their 95% confidence intervals (CI) of cancer in total and of specific cancer types. Results: Patients with schizophrenia (N = 13.711) had a lower incidence of cancer diagnosis compared to those without (N = 68.555). Specifically, 10.4% of patients with schizophrenia and 12.5% of patients without the condition were diagnosed with cancer (*p* < 0.001). Cox regression analysis showed a significant association between schizophrenia and subsequent cancer in the total population (HR: 0.82; 95% CI: 0.76–0.90), and among men (HR: 0.70; 95% CI: 0.61–0.80), but not among women (HR: 0.94, 95% CI: 0.84–1.04). Analyses stratified by cancer type and sex revealed a strong and significant association between schizophrenia and a decreased risk of prostate cancer in men (HR: 0.38; 95% CI: 0.24–0.61). Furthermore, there was also a negative association between schizophrenia and colorectal cancer risk in men, but statistical significance was not reached (HR: 0.58; 95% CI: 0.37–0.93). Conclusions: This study demonstrates negative associations between schizophrenia and subsequent cancer, and more specifically in men for prostate and colorectal cancer. However, further research is required to explore the underlying reasons for these associations.

## 1. Introduction

While the global prevalence of schizophrenia remains below 1%, the absolute number of cases continues to increase due to factors such as population growth and aging [1]. Compared to the general population, individuals with schizophrenia face a significantly reduced life expectancy of 15 to 20 years on average and experience a two to three times higher mortality rate [2,3,4]. Consequently, schizophrenia is considered one of the most severe mental disorders, playing a significant role in the overall burden of disease and economic strain worldwide [5].

Individuals diagnosed with schizophrenia face high mortality rates, largely due to comorbidities and consequences of the disease’s symptoms. Factors such as poor health habits, metabolic disorders, cardiovascular diseases, and co-existing depression influence the mortality risk positively. Also, cognitive and social impairment are known side effects of schizophrenia [6].

The high mortality rates associated with schizophrenia reveal suicide as the most common unnatural cause. However, in terms of natural causes, cancer was shown to be the leading comorbid cause of mortality in individuals with schizophrenia in France at 20.2%, followed by cardiovascular diseases with 17.2% [7]. However, epidemiological research on cancer prevalence in patients with schizophrenia presents conflicting results. Some studies indicate a lower risk of cancer in individuals with schizophrenia, others suggest a higher risk, and yet others find comparable cancer risk between individuals with and without schizophrenia [3].

Given the prevalent cancer-related risk factors in individuals with schizophrenia, such as tobacco consumption, there is, for example, a higher prevalence of lung cancer within this cohort anticipated [8].

This study aims to clarify the potential association between schizophrenia and the incidence of cancer. Specifically, we will evaluate the relationship between schizophrenia and cancer incidence among patients in German general practices from 2005 to 2022. The Disease Analyzer database offers representative data for Germany’s general population to gain reliable information with high level of significance and therefore, to address the existing knowledge gap on the association between schizophrenia and cancer incidence. The necessity of this study is the result of the scientific inconsistencies on this association. It is crucial to clarify the existence and the type of association to ultimately improve health care and management of individuals with schizophrenia.

## 2. Database and Methods

### 2.1. Database

This retrospective cohort study was based on data from the Disease Analyzer database (IQVIA), which contains drug prescriptions, diagnoses, and basic medical and demographic data obtained directly and in anonymous format from computer systems used in the practices of general practitioners and specialists [9]. The database covers approximately 3% of all private practices in Germany. The sampling method for the Disease Analyzer database is based on summary statistics from all physicians in Germany published yearly by the German Medical Association. IQVIA uses these statistics to determine the panel design according to the four strata including specialist group, German federal state, community size category, and age of physician. It has previously been shown that the panel of practices included in the Disease Analyzer database is representative of general and specialized practices in Germany with respect to age, sex and prescriptions [9]. Finally, this database has already been used in previous studies focusing on cancer [10,11,12].

### 2.2. Study Population

**Figure 1:**
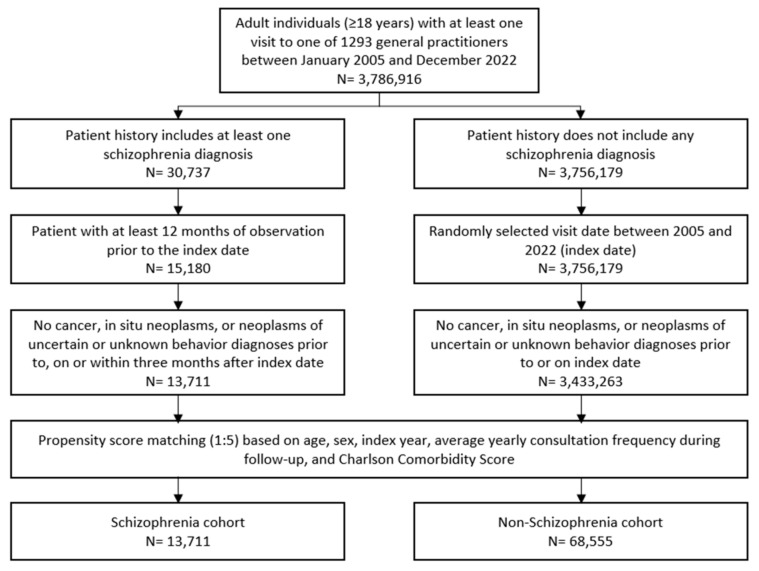
Selection of study patients.

Patients aged 18 and older with a documented schizophrenia diagnosis (ICD-10: F20) in 1293 general practices in Germany between January 2005 and December 2022 were included. A random schizophrenia documentation date was considered as the index date (Figure 1). Patients with diagnoses of cancer (ICD-10: C00–C97), in situ neoplasms (ICD-10: D00–D09), and neoplasms of uncertain or unknown behavior diagnoses (ICD-10: D37–D48) prior to index date, on the index date, or within three months after the index date were excluded. Only patients with an observation time of at least 12 months prior to the index date were included.

Individuals without schizophrenia diagnoses were matched to schizophrenia patients using nearest neighbor propensity score matching (1:5) based on similar inclusion criteria. The matching factors included age, sex, index year, average yearly consultation frequency during follow-up, and Charlson Comorbidity Index (CCI). The CCI is a weighted index used in administrative database studies accounting for the number and severity of comorbidities. It includes a wide range of comorbidities, such as macrovascular diseases, pulmonary diseases, gastrointestinal, liver, and renal diseases, diabetes, AIDS, and others [13]. For the non-schizophrenia cohort, the index date was that of a randomly selected visit occurring between January 2005 and December 2022 (Figure 1).

### 2.3. Study Outcomes

The outcomes of the study were the initial diagnoses of cancer in total (ICD-10: C00–C97), as well as most frequent cancer types including esophagus and stomach (ICD-10: C15, C16), colon and rectum (ICD-10: C18, C20), pancreas (ICD-10: C25), bronchus and lung (ICD-10: C34), malignant melanoma of skin (ICD-10: C43), female breast (ICD-10: C50), female genital organs (ICD-10: C51–C58), prostate (ICD-10: C61), lymphomas (ICD-10: C81–C86) and leukemias (ICD-10: C91–C95) in the up to 10 years following the index date as function of schizophrenia.

### 2.4. Statistical Analyses

Differences in the sample characteristics and diagnosis prevalence between schizophrenia and non-schizophrenia cohorts were compared using the Wilcoxon signed-rank test for continuous variables, the McNemar test for categorical variables with two categories, and the Stuart–Maxwell test for categorical variables with more than two categories.

The 10 year cumulative incidence of cancer in total was further studied with Kaplan–Meier curves. These results were compared using the log-rank test. Finally, a univariate Cox regression analysis was conducted to assess the association between schizophrenia and cancer. These models were conducted separately for the female and the male population. Results from the Cox regression model are presented as hazard ratios (HRs) and 95% confidence intervals (CIs). A *p*-value of <0.001 was considered statistically significant due to multiple comparisons. All analyses were conducted using SAS version 9.4 (SAS Institute, Cary, NC, USA).

## 3. Results

### 3.1. Basic Characteristics of the Study Sample

The present study included 13,711 individuals with and 68,555 without schizophrenia. The basic characteristics of study patients are displayed in Table 1. The mean age was between 52.3 and 52.5 years, 50.2% were female. Patients visited physicians an average 6.9 times per year during the follow-up. 

### 3.2. Cumulative Incidence of Cancer Among Patients With and Without Schizophrenia

**Figure 2:**
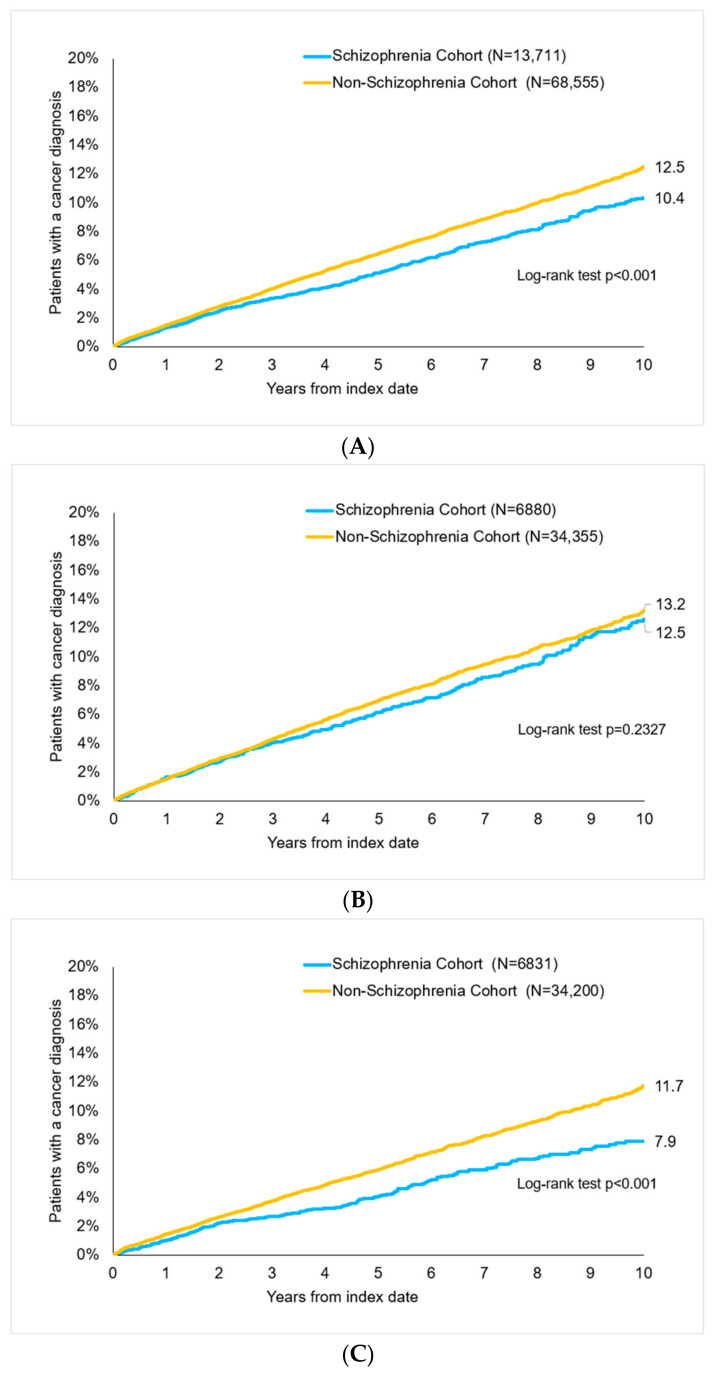
Cumulative incidence of cancer in patients with and without schizophrenia, log-rank test to analyze survival of schizophrenia cohort vs. non-schizophrenia cohort. (**A**) All patients. (**B**) Women. (**C**) Men.

After up to 10 years of follow-up, 10.4% of schizophrenia and 12.5% of non-schizophrenia patients were diagnosed with cancer (*p* < 0.001, Figure 2). There were 12.6% of women with schizophrenia and 13.2% of women without schizophrenia (*p* = 0.233) as well as 7.9% vs. 11.7% of men (*p* < 0.001) with cancer diagnoses (Figure 2).

### 3.3. Association of Schizophrenia with Cancer Diagnoses

In the regression analysis, schizophrenia was significantly negatively associated with a subsequent cancer diagnosis in the total population (HR: 0.82; 95% CI: 0.76–0.90), among men (HR: 0.70; 95% CI: 0.61–0.80) but not among women (HR: 0.94; 95% CI: 0.84–1.04). In women, no significant associations were observed in the stratified analyses by cancer type. In men, schizophrenia was strongly and significantly associated with a decreased prostate cancer risk (HR: 0.38; 95% CI: 0.24–0,61). Moreover, in men there was a strong negative association between schizophrenia and colorectal cancer risk (HR: 0.58; 95% CI: 0.37–0.93), but a *p*-value of <0.001 was not reached (Table 2).

As schizophrenia and non-schizophrenia cohorts were slightly but significantly different in average CCI, a sensitivity analysis using a multivariable regression model adjusted for CCI was conducted. Similar to the main analysis, in this sensitivity analysis, schizophrenia was negatively associated with cancer (HR: 0.83; 95% CI 0.77–0.91).

## 4. Discussion

This study on cancer incidence among patients diagnosed with schizophrenia in German general practices from 2005 to 2022 yielded significant findings. The study sample included two cohorts, one of 13,711 patients with schizophrenia and another cohort of 68,555 patients without the condition. The analyses identified a significant negative association between schizophrenia and the overall cancer risk. Whereas 12.5% of patients not diagnosed with schizophrenia were found to have cancer, it was only 10.4% of patients with schizophrenia who had records of a cancer diagnosis. Stratification by sex resulted in a significant HR only in men, showing a decreased risk for prostate and colorectal cancers. Notably, only the negative association between schizophrenia and prostate cancer risk was statistically significant. In women, no association was shown.

The relationship between schizophrenia and cancer risk has been extensively examined in several studies, returning a variety of, and to some extent contradictory, findings. Our study revealed a notable association between a diagnosis of schizophrenia and a reduced overall risk of developing cancer. This striking pattern does not appear to be influenced by a single factor, instead, it is likely shaped by a multitude of elements.

Among the factors contributing to this reduced cancer risk, certain genes have been identified as potentially associated with a lower incidence of cancer. Huang et al. have shown that there are specific genes common to both schizophrenia and cancer that could potentially inhibit tumor formation [14]. Similarly, Özbey et al. have found that the A1 allele could offer protection against lung cancer [15], while Wang et al. have suggested the XRCC4 gene could act as a protective factor against schizophrenia [16]. These three genes were observed to be more common in individuals diagnosed with schizophrenia than in those without the condition.

In addition, Zhou et al. have concluded that tumor suppressor genes, which have the ability to stop or slow down cell growth and multiplication, could impact the normal development or functioning of neural tissues, thereby potentially leading to schizophrenia [17]. This association is further supported by the observation that not only do individuals with schizophrenia exhibit a lower rate of cancer incidence, but this trend is also seen in their first-degree relatives [18]. The complicated interaction between schizophrenia and cancer, with genes potentially acting as moderators or confounders, warrants further investigation.

Another influential factor to consider is the role of antipsychotic medications, which are the most commonly used treatment for schizophrenia symptoms [19]. These medications justify a closer examination to determine their potential impact on the relationship between schizophrenia and the chance of a cancer diagnosis. While a nested case-control study by Taipale et al. has reported a significant association between long-term exposition to prolactin-increasing antipsychotics and increased odds of breast cancer [20], other research suggests that antipsychotic drugs overall may hold potent anti-cancer properties. In particular, these medications have been shown to not only inhibit the growth and spread of tumors, but also to induce the death of cancer cells [21,22,23]. However, more research is needed to fully understand this complex relationship.

There has been a third influential factor detected in individuals with schizophrenia that potentially could be linked to lower cancer risk, as suggested by Raviv et al. [24]. This factor lies in the comparatively low level of sexual activity amongst individuals with schizophrenia. According to Cournos et al. and Yang et al. this reduced sexual activity is attributable to several factors [25,26]. In a systematic review, Meade and Sikkema found that individuals with severe mental disorders, including schizophrenia, have fewer sexual partners than the general population [27]. Furthermore, research by Grabovac et al. indicates a positive correlation between the number of sexual partners and the odds of developing cancer [28]. Therefore, it is reasonable to suggest that the lower numbers of sexual partners in individuals with schizophrenia could be an influential factor or a confounding variable to the lower incidence of cancer observed in this population. However, more research is needed to confirm this hypothesis.

In general, individuals with schizophrenia show significant medical comorbidity [29] and are also known to have a relatively high prevalence of cancer-related risk factors. Those include tobacco consumption, alcohol dependence, obesity, lack of physical exercise and unhealthy diet [30]. Against our expectations and despite the fact that patients with psychiatric disorders are often heavy smokers [8], there was no association shown between schizophrenia and lung cancer risk in our study, although we did not control for tobacco smoke.

Even though our study indicates that individuals with schizophrenia, especially men, are less likely to develop certain types of cancer such as prostate and colorectal cancer compared to the general population, it is crucial to understand that this population is still vulnerable when it comes to cancer. They are confronted with a higher mortality rate, with a pooled standardized mortality ratio of 1.4 [30] and a worse prognosis for cancer [4,31,32]. It is a consequence of factors including unhealthy lifestyle factors, poor overall health with comorbidities like cardiovascular diseases and depression, side effects of antipsychotic medication, suicide, and inadequate medical care. The inadequate medical care comprises screening, diagnosis, and treatment [33]. Individuals with psychotic disorders, for instance, are 22% more likely to have metastases at the time of diagnosis compared to those without psychotic disorders [34]. Moreover, nearly half of the patients with schizophrenia also suffer from major depressive disorder, which is often poorly screened and treated due to its symptoms being confused with the negative symptoms of schizophrenia [35].

The instances highlighted above underscore the complexity of schizophrenia’s clinical profile. As such, healthcare professionals working with individuals with schizophrenia need to be aware of these unique risk factors. There is a pressing need for public health initiatives to focus on targeted interventions to enhance cancer screening and treatment in patients with schizophrenia. The discussed associations are potential factors, and more research is needed to fully understand these relationships and the protective factors against cancer in patients with schizophrenia. This knowledge could possibly also be applied to other populations to aid in the prevention and treatment of cancer.

## 5. Limitations

The present study has several limitations that warrant acknowledgment. Firstly, the retrospective design generally has drawbacks due to the reliance on pre-existing data, which was not collected with the specific research question in mind. This means that the study could not control for lifestyle factors such as tobacco or alcohol consumption, nor does it provide information on medication use, family status, or mortality, as the Disease Analyzer does not include these variables. Additionally, the reliance solely on the ICD-10 classification for diagnoses and co-diagnoses means that no information was available on the disease severity of patients with schizophrenia. Furthermore, the dataset only includes information from an outpatient setting, which limits the transfer to inpatient data. Finally, since patients are only visible in one specialty group and cannot be tracked across other specialties or inpatient settings, records of diagnoses from other settings cannot be avoided.

## 6. Conclusions

This study demonstrates negative associations between schizophrenia and subsequent cancer, and more specifically in men for prostate and colorectal cancer. Despite the observed lower incidence of cancer among individuals with schizophrenia, this population remains particularly vulnerable to cancer due to a higher mortality rate that is influenced by numerous factors such as schizophrenia-specific symptoms and comorbidities. Therefore, further research is required to explore the underlying reasons for the associations observed in this study.

## Figures and Tables

**Table 1:** Baseline characteristics of the study sample (after 1:5 propensity score matching).

Variable		Patients with Schizophrenia (N, %)	Patients Without Schizophrenia (N, %)	*p*-Value ^1^
		N = 13,711	N = 68,555	
Age (in years)	Mean (SD)	52.3 (18.0)	52.5 (17.9)	0.498
	18–40	3945 (28.8)	19.480 (28.4)	0.322
	41–50	2513 (18.3)	12.233 (17.8)	
	51–60	2786 (20.3)	13.933 (20.3)	
	61–70	2012 (14.7)	10.222 (14.9)	
	>70	2455 (17.9)	12.687 (18.5)	
Sex	Female	6880 (50.2)	34.355 (50.1)	0.888
	Male	6831 (49.8)	34.200 (49.9)	
Annual physician visits during follow-up period	Mean (SD)	6.9 (4.7)	6.9 (4.7)	0.127
Charlson Comorbidity Index (CCI)	Mean (SD)	1.6 (1.9)	1.5 (1.8)	0.003
	CCI 0	5267 (38.4)	26.706 (39.0)	0.003
	CCI 1	2992 (21.8)	15.376 (22.4)	
	CCI 2	2169 (15.8)	10.995 (16.0)	
	CCI >2	3283 (24.0)	15.478 (22.6)	
Index year	2005–2007	1028 (7.5)	5149 (7.5)	0.809
	2008–2010	1182 (8.6)	6092 (8.9)	
	2011–2013	1830 (13.4)	9053 (13.2)	
	2014–2016	2526 (18.4)	12.357 (18.0)	
	2017–2019	3395 (24.8)	17.011 (24.8)	
	2020–2022	3750 (27.3)	18.893 (27.6)	

Proportions of patients in N, % given, unless otherwise indicated. SD: standard deviation. ^1^ Wilcoxon signed-rank test for continuous variables, McNemar test for categorical variables with two categories, and the Stuart–Maxwell test for categorical variables with more than two categories.

**Table 2:** Association between schizophrenia and subsequent cancer diagnosis in patients seen by general practitioners in Germany.

	All Patients		Women		Men	
Cancer Diagnosis	HR (95% CI)	*p*-Value ^1^	HR (95% CI)	*p*-Value ^1^	HR (95% CI)	*p*-Value ^1^
Cancer total	0.82 (0.76–0.90)	<0.001	0.94 (0.84–1.04)	0.233	0.70 (0.61–0.80)	<0.001
Esophagus/stomach	0.70 (0.43–1.15)	0.162	0.54 (0.23–1.26)	0.152	0.83 (0.45–1.52)	0.538
Colon/rectum	0.85 (0.63–1.13)	0.260	1.15 (0.79–1.67)	0.463	0.58 (0.37–0.93)	0.023
Pancreas	0.77 (0.44–1.32)	0.338	0.66 (0.30–1.45)	0.298	0.90 (0.42–1.91)	0.775
Bronchus/lung	0.88 (0.66–1.18)	0.392	0.94 (0.62–1.43)	0.779	0.83 (0.56–1.24)	0.365
Skin	0.60 (0.34–1.04)	0.070	0.70 (0.35–1.41)	0.318	0.47 (0.19–1.19)	0.112
Lymphomas	0.90 (0.59–1.35)	0.596	0.98 (0.57–1.67)	0.933	0.80 (0.42–1.51)	0.482
Leukemias	0.60 (0.32–1.11)	0.105	0.42 (0.15–1.16)	0.094	0.79 (0.35–1.76)	0.563
Prostate					0.38 (0.24–0.61)	<0.001
Female breast			0.88 (0.69–1.12)	0.293		
Female genital organs			1.17 (0.83–1.67)	0.371		

^1^ univariate Cox regression analysis with likelihood chi-square statistic. HR: Hazard Ratio. CI: confidence interval.

## Data Availability

The data and the code used for this study are available from the corresponding author upon reasonable request.
